# Neurod2 knockdown in *Xenopus laevis* tadpole brain retains cells in a proliferating, progenitor-like state

**DOI:** 10.17912/micropub.biology.002018

**Published:** 2026-02-23

**Authors:** Caroline W Beck, Sulagna Banerjee, Robert C Day

**Affiliations:** 1 Department of Zoology, University of Otago, Dunedin, OTA, NZ; 2 Department of Biochemistry, University of Otago, Dunedin, OTA, NZ

## Abstract

Neurogenic differentiation factor 2, encoded by the NEUROD2 gene, is a proneural transcription factor required for neuronal differentiation and survival. Haploinsufficiency of NEUROD2 can cause neurodevelopmental disorders with or without seizures in human infants and causes spontaneous seizures in *Xenopus* tadpoles. We compared transcriptomes of whole brains dissected from F _0_ *neurod2 ^-/-^* (mosaic) stage NF47 *Xenopus laevis* tadpoles to those of control siblings. *neurod2* knockdown increased expression of cell cycle-associated genes and decreased nerve growth factor (NGF) and chromatin modifying genes. Our results suggest Neurod2 deficiency prevents neural progenitor cells exiting the cell cycle and differentiating, predisposing the brain to hyper-excitability.

**Figure 1. Differential gene expression and gene ontology analysis of Neurod2 CRISPant tadpole brains.**
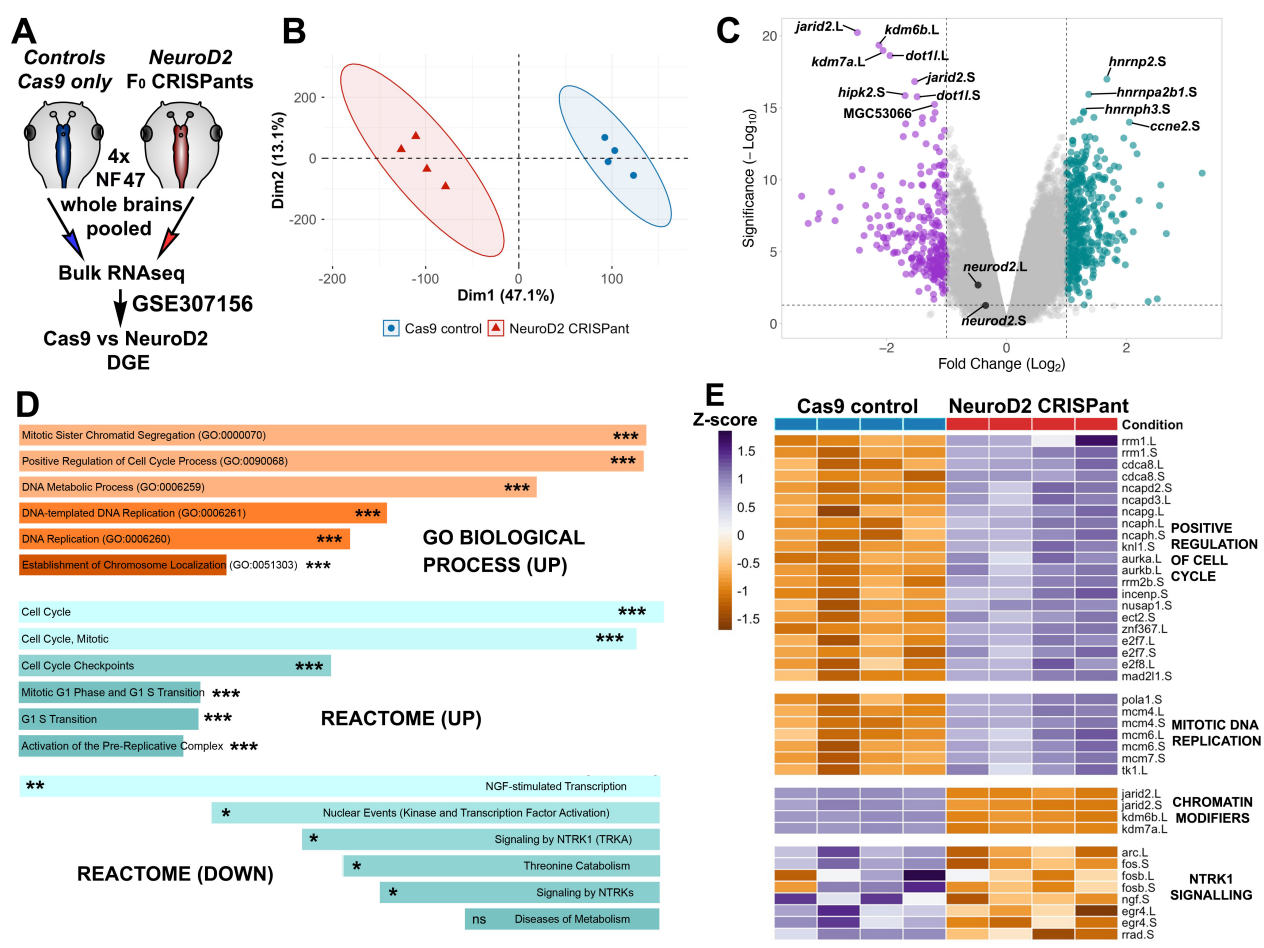
**A)** Schematic of sample preparation and processing. Each sample is four pooled stage NF47 tadpole brains, all animals were from a single sibship. **B)** Unsupervised PCA plot of normalised read counts (Log _2_ CPM) of CRISPant (red triangles) vs. control (blue circles) brain samples. Ellipses enclose 95% confidence intervals for each group. **C)** Volcano plot with top 12 significantly changed genes labelled (ranked by Manhattan). The *neurod2* .L and .S homeologues are also labelled. 70 up regulated (teal) and 75 down-regulated (purple) genes >Log _2_ FC 1.0 and p<0.01. **D).** Up-regulated genes with LFC >1 were compared to a custom background list of tadpole stage NF47 brains, the top 6 enriched categories are shown. PAdj (FDR) *** <0.001, **<0.01, *<0.05, ns non-significant for GO Biological Process and Reactome 2024 pathway, using Enrichr. E) Heat maps of selected enriched category genes, colour based on z-score of normalised Log _2_ CPM, as indicated on the key.

## Description

**Description**

NEUROD2 encodes a basic helix-loop-helix transcription factor that can induce cell cycle arrest and differentiation of neurons (Messmer et al., 2012; Olson et al., 2001). Two missense variants in the DNA-binding domain cause developmental and epileptic encephalopathy 72 (DEE72:&nbsp; OMIM #618374) in human infants and induce spontaneous seizures in *Xenopus* tadpoles&nbsp; (Sega et al., 2019). NEUROD2 variants carrying these mutations could not induce ectopic neurons in early *Xenopus* embryos, linking the proneural role of the gene to seizure susceptibility. Since this initial discovery, other missense mutations in NEUROD2 have been linked to developmental delay without seizures (Mis et al., 2021; Politano et al., 2023; Runge et al., 2021).

We have previously shown that mosaic CRISPR knockdown of *neurod2* in F _0 &nbsp;&nbsp;_ *Xenopus laevis* tadpoles leads to spontaneous seizures with telltale signs of inflammation such as a leaky blood brain barrier (Banerjee et al., 2024). The sgRNA we used results in a 4 bp deletion that truncates the protein early in the DNA binding domain, resulting in *neurod2* ^-/-^ (mosaic) tadpoles with 25-30% overall editing levels as determined from the pooled samples. Protein levels were not determined in this study. Here, we have investigated the transcriptional changes in the brain by comparing *neurod2* ^-/-^ (mosaic) CRISPants with their siblings that were injected only with Cas9.

A schematic of the experimental design can be found in panel A. The transcriptomes of brains from *neurod2* ^-/-^ (mosaic) tadpoles clustered separately from sibling controls in an unsupervised PCA plot of all normalised read counts (Panel B). Differential gene expression analysis revealed 407 up- and 254 down-regulated genes (>2-fold change and pAdj < 0.05). Genes with the 12 top ranked scores can be seen in the labelled Volcano plot (Panel C). Three heterogeneous nuclear ribonucleoproteins (hnRNPs): the S homeologues of *hnrnpr* , *hnrnpaB1* and *hnrnph3* , were all highly upregulated in CRISPant brains. HnRNPs bind to nascent transcripts (pre-mRNA) to regulate their transport and splicing, so this result suggests reduced Neurod2 leads to increased post-transcriptional control. Dis-regulation of hnRNPs has recently been associated with a broad range of brain disorders (reviewed in (Brandão-Teles et al., 2024), and variants in the human ortholog HNRNPR are linked to neurodevelopmental delay (Duijkers et al., 2019; Gillentine et al., 2021).

The *neurod2* ^-/-^ (mosaic) brains showed an overall 21% reduction in *neurod2.S* and 28% for *neurod2.L,* consistent with observed editing levels. Reduced *neurod2* correlated with significant up-regulation of many genes involved in control of the cell cycle *. Ccne2* , one of the most significantly up-regulated genes (Panel C, 4-fold change), encodes Cyclin E2, which partners with Cdk2 to drive G1 to S-phase transition. Up-regulation of genes associated with cell cycle regulation was also a strong signature of gene enrichment analyses (Panel D).&nbsp; Heatmaps of z-scores for GO:0090068 “Positive Regulation of Cell Cycle Process” (PAdj=7.9E-10) and GO:1902969 “Mitotic DNA Replication” (PAdj=5.4E-05) show consistent up-regulation of these sets within samples (Panel E), suggesting this is linked to editing levels in the brain. The relatively higher expression of cell cycle genes in *neurod2* ^-/-^ (mosaic) brains compared to their sibling controls suggests that more neural progenitor cells are present, and that fewer are exiting the cell cycle to differentiate. Further, when we looked at cell type markers identified in a previous transcriptome analysis of *X. laevis* brains (Ta et al., 2022) we found that CRISPants express lower levels of neuronal and glial markers, and higher levels of neural progenitor cell (NPC) markers (Table 1).

Intriguingly, the seven most significantly down-regulated genes (Panel C) all have roles in chromatin remodelling. Jarid2 protein helps recruit polycomb repressive complex2 (PRC2) to target genes. In humans, JARID2 is expressed in neurons and haploinsufficiency is associated with developmental delay and ID (Verberne et al., 2021). Two lysine demethylase coding genes *kdm6b* .L, and *kdm7a* .L, are also highly downregulated in CRISPant brains which points to an increase in repressive H3 methylation groups. Dot1l (disruptor of telomeric silencing like 1) is an H3K79 histone methyltransferase with a role in regulating cell cycle (Kim et al., 2014); in developing mouse brains it promotes progenitor proliferation (Franz et al., 2019). Very recently, partial loss of DOT1L has been shown to result in a neurodevelopmental disorder, with altered neuronal transcription and behavioural responses in mouse and zebrafish models (Maroni et al., 2025). Finally, *hipk2* .S encodes homeodomain interacting protein kinase 2, and targets of this enzyme include histone 2B, although this role is linked to cytokinesis rather than DNA accessibility. HIPK2 is highly expressed in human central nervous system, and in mice it promotes cortical neurogenesis (Sardina et al., 2023).

Gene ontology enrichment analysis of down-regulated genes revealed that Nerve growth factor (NGF)-stimulated transcription and signalling by NTRK1 (Reactome, pAdj=0.005 and 0.035, panel D) were over-represented. NGF is the ligand for Ntrk1, a receptor tyrosine kinase, and the pathway regulates neuronal survival and axon guidance. In *neurod2* ^-/-^ (mosaic) tadpole brains, the *ntrk1* .L and S homeologues are expressed at ~80% of control levels, indicating only a small reduction in the transcription of the receptor. In contrast, *ngf.S* &nbsp;expression is 2.3-fold lower in *neurod2* ^-/-^ (mosaic) brains, and several downstream targets are also reduced, suggesting suppression of this pathway in the CRISPant brains (Panel E). While no links have been found between this pathway and DEE, missense mutants in the related gene NTRK2 are known to cause DEE58 (OMIM #617830: Hamdan et al., 2017).

Taken together, the observed up-regulation of cell cycle genes, likely increase in histone methylation-based repressive marks and down-regulation of neurotrophic signalling suggests *neurod2* ^-/-^ (mosaic) brains have a greater proportion of cells that fail to exit the proliferating progenitor state. While our ontology analysis assumes that human genes and their *X. laevis* orthologues have conserved roles in brain development, modelling of rare genetic disease in *Xenopus* indicates that fundamental processes are conserved (Willsey et al., 2024). Our analysis of *neurod2* ^-/-^ (mosaic) brain transcriptomes, while limited to a single stage, shows that tadpole models may be useful in understanding the aetiology of other DEE, providing a useful step on the way to future therapy.

**Table 1: Significantly changed cell-type marker expression in neuroD2 ^-/-^ (mosaic) tadpole brains. Cell type designation from (Ta et al., 2022), NPC are neural precursor cells.**

**Table:** 

**Gene name**	Gene ID (NCBI)	**LogFC**	**PAdj**	**Cell Type**	**Change in CRISPants**
*notch1.S*	394367	0.525	5.27E-07	NPC	UP
*vim.L*	386601	0.521	8.44E-07	NPC	UP
*nes.S*	397776	0.728	0.00098	NPC	UP
*hes5.2.L*	396151	0.454	0.00028	NPC	UP
*lmx1b.2.L*	108710365	-0.683	8.44E-07	Neurons	DOWN
*mpz.S*	100037196	-1.457	0.00080	Glia	DOWN
*aif1l.L*	444577	-0.437	0.00442	Glia	DOWN
*mbp.S*	399102	-0.284	0.00105	Glia	DOWN
*sox10.L*	398422	-0.352	0.03130	NPC+Glia	DOWN
*glul.S*	444294	-0.859	1.17E-07	Glia + Neurons	DOWN
*glul.L*	398556	-0.893	1.02E-07	Glia + Neurons	DOWN

## Methods

F _0_ CRISPants were made using a sgRNA that targets both L and S homeologues of *Xenopus laevis* NeuroD2, in the DNA binding domain, resulting formation of a non-functional protein (Banerjee et al., 2024). Whole brains were dissected at Nieuwkoop and Faber (NF) stage 47 (Nieuwkoop & Faber, 1994) and snap frozen on dry ice in groups of four. Four such biological replicate samples were used for each condition. Total RNA was extracted using a Qiagen RNeasy mini kit with an on-column DNaseI treatment. Libraries were prepared using 1 mg of RNA with RIN >9.5 and sequenced using Illumina Nextseq P2 by the Otago Genomics Facility, University of Otago, New Zealand, generating ~30 M reads per sample. Single reads of 100 bp were mapped to XENLA_10.1 genome build (Xenbase, accessed November 2024, RRID:SCR_003280) using DRAGEN v 4.0.4. Galaxy EU (RRID:SCR_006281) was used to generate read counts using FeatureCounts (Liao et al., 2014) and to normalise read counts and identify differentially expressed genes with EdgeR (Robinson et al., 2010). Sequencing data and read counts are available at NCBI GEO (Edgar et al., 2002, RRID:SCR_005012) under accession GSE307156. Genes with fold change of > 2 and Benjamini-Hochberg corrected FDR <0.05 (PAdj) between control and CRISPant brains were considered differentially expressed. Enrichr (RRID:SCR_001575) was used with a custom background list of brain expressed genes to identify enriched gene ontologies (Chen et al., 2013; Kuleshov et al., 2016). GO Biological Process (Ashburner et al., 2000; The Gene Ontology et al., 2023) and Reactome 2024 (Milacic et al., 2023) tables were mined for this analysis. The Volcano plot was created using VolcaNoseR (Goedhart & Luijsterburg, 2020). Heatmaps were made from Z-scores of normalised read counts using the pheatmap package (Kolde, 2025) in R v4.5.1 (R Development Core Team, 2024). Principal component analysis (PCA) plot was also generated in R v4.5.1 from normalised read counts (Log _2_ counts per million) using FactoMineR (Lê et al., 2008) and factoextra packages (Kassambara & Mundt, 2020) and plots rendered with ggplotR (Wickham, 2016).

**Animal Ethics** : all work with *X. laevis* was approved by the University of Otago Animal Ethics Committee under AUP 22/12 and AUP 22/24.
